# Genome-wide investigation identifies a rare copy-number variant burden associated with human spina bifida

**DOI:** 10.1038/s41436-021-01126-9

**Published:** 2021-03-08

**Authors:** Paul Wolujewicz, Vanessa Aguiar-Pulido, Alice AbdelAleem, Vidya Nair, Gaurav Thareja, Karsten Suhre, Gary M. Shaw, Richard H. Finnell, Olivier Elemento, M. Elizabeth Ross

**Affiliations:** 1 Center for Neurogenetics, Brain and Mind Research Institute, Weill Cornell Medicine, New York, NY USA; 2 Neurogenetics Research, Weill Cornell Medicine Qatar, Doha, Qatar; 3 Department of Physiology and Biophysics, Weill Cornell Medicine–Qatar, Doha, Qatar; 4 Pediatrics, Stanford University School of Medicine, Stanford, CA USA; 5 Center for Precision Environmental Health, Departments of Molecular and Cellular Biology, Molecular and Human Genetics and Medicine, Baylor College of Medicine, Houston, TX USA; 6 Department of Physiology and Biophysics, Weill Cornell Medicine, New York, NY USA; 7 The HRH Prince Alwaleed Bin Talal Bin Abdulaziz Alsaud Institute for Computational Biomedicine, Weill Cornell Medicine, New York, NY USA; 8 Caryl and Israel Englander Institute for Precision Medicine, Weill Cornell Medicine, New York, NY USA

## Abstract

**Purpose:**

Next-generation sequencing has implicated some risk variants for human spina bifida (SB), but the genome-wide contribution of structural variation to this complex genetic disorder remains largely unknown. We examined copy-number variant (CNV) participation in the genetic architecture underlying SB risk.

**Methods:**

A high-confidence ensemble approach to genome sequences (GS) was benchmarked and employed for systematic detection of common and rare CNVs in two separate ancestry-matched SB case–control cohorts.

**Results:**

SB cases were enriched with exon disruptive rare CNVs, 44% of which were under 10 kb, in both ancestral populations (*P* = 6.75 × 10^−7^; *P* = 7.59 × 10^−4^). Genes containing these disruptive CNVs fall into molecular pathways, supporting a role for these genes in SB. Our results expand the catalog of variants and genes with potential contribution to genetic and gene–environment interactions that interfere with neurulation, useful for further functional characterization.

**Conclusion:**

This study underscores the need for genome-wide investigation and extends our previous threshold model of exonic, single-nucleotide variation toward human SB risk to include structural variation. Since GS data afford detection of CNVs with greater resolution than microarray methods, our results have important implications toward a more comprehensive understanding of the genetic risk and mechanisms underlying neural tube defect pathogenesis.

## INTRODUCTION

Neural tube defects (NTDs) are anomalies of the central nervous system (CNS) present at birth that manifest with varying subtypes and severity and are among the most common structural birth defects. In more severe NTD subtypes the rostral neural tube fails to close, exposing brain (anencephaly) or brain and cervical–thoracic spine (craniorachischisis), resulting in intrauterine or neonatal death. In contrast, spina bifida aperta (SB, myelomeningocele) is a neural tube closure defect most often confined to the caudal spine below the level of T10. With advances in surgical repair and management, the majority of spina bifida patients will live into adulthood, but will experience lifelong physical challenges including paralysis, associated hydrocephalus requiring cerebrospinal fluid (CSF) shunting, autonomic dysfunction, orthopedic issues, and more. With heritability estimates as high as 70%,^1^ NTDs are thought to arise through an interplay of multiple gene–gene interactions determining genetic predisposition and environmental factors that tip the balance toward failed neurulation.^2^

The successes of folic acid supplementation for prevention has led to an emphasis in genetic and epidemiological NTD research on the disease association with candidate genes involved in folate metabolic pathways. Moreover, it has prompted numerous studies in animals of genes involved in one-carbon metabolism and their link with structural birth defects. In addition, genetically engineered animal models of NTD have revealed the importance to neurulation of signaling pathways such as Wnt/planar cell polarity (PCP),^3^ sonic hedgehog (Shh),^4^ and protein kinase A (PKA).^5^ Mouse models of NTDs have established more than 250 genes whose variants predispose to NTD in the mouse, often showing incomplete penetrance that would suggest additional factors in the genetic background or fetal environment are required for NTD to be manifested.^6^ These insights have spurred a number of candidate gene searches among affected patients and sometimes parents in an attempt to identify genetic variants that confer risk for developing an NTD. Nevertheless, there remains a translational challenge to reconcile mouse and human NTD data in order to pinpoint genes, pathways, and eventually discern patterns of genetic variation that pose NTD risk in humans.

Despite years of clinical studies and investigations in animal models, the patterns of human genetic variation that predispose to NTD remain elusive, limiting our efforts to clearly define the genetic architecture underlying the etiology of NTDs. This may in part be attributed to the relatively small effect sizes of individual genes and the narrow focus of investigations on variation within protein-coding regions of the genome. Genome sequencing (GS) data coupled with recent advances in algorithmic detection of genomic variation offer opportunities to interrogate underexplored forms of potential NTD risk such as structural variants (SVs), whose effects on NTD risk are not well understood. SVs have been shown to alter the structure and dosage of many genes and rare SVs may exert stronger effects on gene expression compared with rare single-nucleotide polymorphisms (SNPs). Rare SVs may also ablate exons and create gene fusions, affecting downstream functionality independent from gene expression. Efforts thus far to interrogate SVs and copy-number variants (CNVs) in NTD cases have largely relied on array-based platforms or exome sequencing (ES). Using those tools, deletions in several genes involving cilia and proteoglycans have been implicated in NTD risk.^7^ Therefore, we hypothesize that novel NTD risk genes and mechanistic insight will be gained from a comprehensive genome-wide evaluation of CNVs from GS data.

We report our first in a series of SV analyses of GS data from two separate ancestry-matched human SB case–control cohorts, focusing on the landscape of high-confidence, likely gene disrupting CNVs. For complex diseases including NTDs, it is important to account for population genetic differences to avoid confounding effects of ancestral variation; thus, we sought to maintain a representative balance of population admixtures in each of our cohorts. In this work, we benchmarked an ensemble of computational tools for CNV detection that was then employed to characterize both common and rare CNVs in well-defined human SB cohorts with the objective of further defining genomic risk contribution in these CNS anomalies.

## MATERIALS AND METHODS

### Study cohorts and genome sequencing

SB subjects who displayed nonsyndromic myelomeningocele were selected as cases for this study. Of the 140 SB cases, 67 were collected in the United States and 73 from Qatar. The US cohort included an additional 46 unrelated controls with another 30 control subjects obtained from the Pan-Cancer Analysis of Whole Genomes (PCAWG) consortium.^8^ These additional 30 germline samples were analyzed and confirmed to maintain similar admixtures as the US cases. The Qatar cohort included an additional 107 unrelated individuals from the same geographic region and who, again, displayed a population admixture similar to cases. In all, the US cohort comprised 143 individuals and the Qatar cohort included 180 individuals. Altogether, 323 anonymized subjects were included in our study encompassing the two populations. The presence of Y-chromosome markers in individuals was used to determine sex ratios in our case–control cohorts. The male:female ratio within in our case group is 0.69 and is 0.68 within our control group, indicating no sex bias in our study.

Genomic DNA was extracted from de-identified infant blood spot cards obtained from the California Genetic Diseases Screening Program as well as from venipuncture samples collected from subjects participating in the national Spina Bifida Clinic at Hamad Medical Corporation in Qatar. DNA extraction was done using the Puregene DNA Extraction Kit (Qiagen, Valencia, CA) and all DNA samples were submitted for GS using an Illumina HiSeq2500 platform to yield short insert paired end 2×100 bp reads. Ancestry-aware study cohorts were obtained by extracting relevant loci via PLINK (v.1.9)^9^ to calculate specific admixture components, and each cohort constituted a representative admixture balance for both cases and controls.

### Alignment, SNV calling, and preprocessing

FASTQ reads were aligned to reference genome hg38 using BWA.^10^ After reads were sorted and duplicates were removed, SNV and indel calling was performed with GATK4 and joint genotyping was carried out on the whole cohort according to GATK Best Practices recommendations.^11^ Only variants with a “PASS” in the filter column were retained. SAMtools^12^ was used on individual bam files to run quality control measures and to assess read depth uniformity. Read depth statistics were also employed in SAMtools or GATK. The median insert size for samples included in the analysis was 413 bp.

### CNV and MEI detection

To maximize high-quality CNV detection from short-read GS data, we deployed numerous callers known to perform well individually and integrated their results into a consensus framework that we optimized and packaged as an ensemble approach (Supplementary Figure 1). Manta (v.1.4),^13^ Delly (v.0.7.7),^14^ Lumpy (v.0.2.13),^15^ CNVnator (v.0.3.3),^16^ and ERDS (v.1.1)^17^ were each employed on individual sample bam files. ERDS also used SNV calls as input for each individual to refine CNV breakpoints. The CNVs detected using the read depth tools (CNVnator and ERDS) were only kept if there was an agreement between calls from each tool, which we defined as those CNVs that are of the same type and that have breakpoints located within close proximity of each other (<2 kb for CNVs<100 kb; <5 kb for CNVs >100 kb). Only calls ≥ 1 kb were retained given the limited ability of these read depth methods to capture smaller CNVs. We added to this set of CNVs the consensus calls made from the tools that also utilize split-read and read pair signatures (Manta, Lumpy and Delly). A consensus call required two of these three tools to agree on the CNV type and that individual breakpoints were located within 1 kb of each other. Only calls ≥300 bp and ≤100 kb were retained, since CNVs outside of this range are prone to yield false positives. MELT (v. 2.0.5)^18^ was used for detection of mobile element insertions, namely Alu, SVA, and L1 elements.

To ensure against software performance dependencies across study cohorts, our ensemble CNV detection approach was implemented as a Docker image and run on individual subjects, generating a single consensus VCF for each sample. Sample VCFs were merged across all samples via SURVIVOR^19^ resulting in a nonredundant set of high-quality candidate CNVs.

### SV annotation and filtering repetitive and low-complexity regions

Each CNV with any predicted overlap with any coding sequencing of the canonical transcript of 20,246 protein-coding genes was annotated as coding. Deletions were considered loss-of-function if they overlapped any coding sequence and duplications were considered loss-of-function if they affected an exon without extending outside the transcript boundaries. Duplications were considered to be copy gain if they spanned the entirety of a transcript. AnnotSV (v.2.0)^20^ was used for annotation of VCF using reference genome hg38. We filtered those CNVs from our call sets that had >70% reciprocal overlap with repetitive and low-complexity regions, which may confound genomic variant detection. As a comprehensive set of repetitive and low-complexity regions, we combined four data sets: (1) the set of assembly gaps defined by University of California–Santa Cruz (UCSC), including centromeres, telomeres, constitutive heterochromatin domains, gaps between or within clones and contigs, and the repeat-dominated short arms of chromosomes 13, 14, 15, 21, and 22; (2) the UCSC list of segmental duplications; (3) the pseudoautosomal regions of the sex chromosomes; and (4) repeat regions as defined by RepeatMasker.^21^

### Benchmarking and CNV simulation analyses

To select the optimal combination of software and parameters included in our ensemble CNV approach, we conducted a number of benchmarking analyses deploying numerous detection algorithms on both real and simulated genomes. We utilized the well-characterized HG002 genome for benchmarking deletion calls obtained from the Genome in a Bottle (GIAB) consortium,^22^ which provides tier 1 benchmark regions of high-quality deletions that we utilized as our ground truth data set. For our simulation data, we used RSVsim to simulate deletions and duplications of a range of sizes at various genomic coordinates. Wgsim in the SAMtools package was used to create comparable GS reads similar to our study cohorts for further benchmarking and analysis. For sensitivity and precision evaluations, we used Truvari for HG002 deletion benchmarking and in-house scripts for accuracy metrics on simulated data.

### Population genomic databases and BAM confirmation

We utilized the following population reference databases to extract population allele frequencies for our detected CNVs: 1000 Genomes Project,^23^ Database of Genomic Variants,^24^ gnomAD-SV.^25^ If the coordinates for a given population reference were relative to reference assembly GRCh37/h19, then they were converted using the UCSC Batch Coordinate Conversion (LiftOver) tool.

To manually assess and validate rare coding CNVs, Integrative Genomics Viewer^26^ and Samplot^27^ were used to visually compare the read depth of the CNV with that of the surrounding regions. This manual curation entailed examining deviations in read depth corresponding to the predicted change in copy number; that is, a 50% reduction for a heterozygous deletion or a 50% increase for a heterozygous duplication. Predicted CNVs were required to have unambiguous start and end breakpoints, which was refined using split-read and/or read pair information.

### Pathway and statistical analyses

Ingenuity Pathway Analysis (IPA), Webgestalt,^28^ and GeneAnalytics^29^ were used to investigate the genes affected by rare coding CNVs and both cases and controls. We utilized the IPA software to identify the top canonical pathways associated with our data set, which consisted of the genes impacted by rare coding CNVs in our SB cases. We also conducted a pathway overrepresentation analysis with the KEGG functional pathway database, considering only protein-coding genes perturbed by rare CNVs since our aim was to assess the impact of coding CNVs. For the burden analyses, we applied two-sided Wilcoxon rank-sum tests to analyze the distributions of common and rare CNVs as well as mobile element insertions (MEIs) in our SB cohorts. Significance of differences between cases and controls in mean values for the number of rare coding CNVs per genome was assessed in each population cohort using two-tailed Student *t*-tests.

### Real-time quantitative PCR

Select rare coding CNVs identified in our cohorts were validated using real-time quantitative polymerase chain reaction (PCR) in samples for which DNA remained available after GS. DNA from four separate individuals that harbored a rare coding CNV was amplified using primers designed to hybridize in the region containing the putative CNV. Fold changes of expression were calculated and compared with the average value of three control samples, which contained two copies of the gene-specific region in which primers were designed. All reactions were performed in technical triplicates and ß-actin was used as an internal control. Fold changes of expression were calculated using the 2^−ΔΔCT^ method and the gene-specific primers used for four CNVs are listed in Supplementary Table 2.

## RESULTS

### Cohort characteristics and CNV workflow

**Fig. 1:**
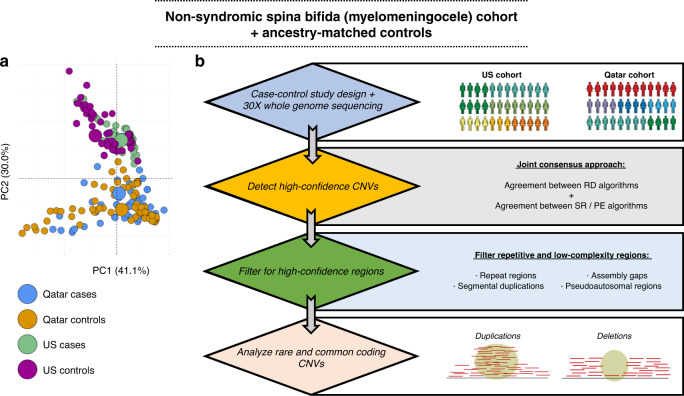
Spina bifida cohorts and analytical workflow. (**a**) Two first principal components (PCs) from population admixture data of the individuals in the study colored by cohort and case status. (**b**) Study design and approach for high-confidence copy-number variant (CNV) detection in reliable genomic regions using genome sequencing data. PE paired end, RD read depth, SR split-read.

Two separate ancestry-matched cohorts (US and Qatar) were subjected to GS and were analyzed in this study comprising a total of 323 subjects. The US cohort included 67 cases and 76 controls and the Qatar cohort included 73 cases and 107 controls. Cases in each cohort had a clinical diagnosis of nonsyndromic SB and controls were balanced with similar ancestry admixture components as the cases in each study cohort. Principal component analysis (PCA) of the population admixtures for each cohort show that the cases and controls are comprised of similar ancestral backgrounds (Fig. 1a).

We devised an optimized methodology for CNV detection featuring a consensus strategy that utilizes five CNV/SV callers as well as input from SNV detection for call refinement. This ensemble approach leveraged multiple genomic signatures into a joint consensus integrative framework (Supplementary Figure 1). The approach employed a combination of read depth callers that performed among the best with regard to accuracy metrics in benchmarking studies and our own analyses.^30^ Our approach also incorporated split-read and discordant read pair evidence from several tools to yield high-quality deletions and duplications >300 bp. As part of initial analyses, our method was benchmarked against the well-characterized HG002 genome from the GIAB consortium^22^ yielding an F1-measure = 82.53 in calling deletions. Since this benchmarking data set does not include tandem duplications or copy-number gains, results were further refined and tested on simulated genomes with 30× coverage harboring a number of duplications of various sizes. These initial benchmarking efforts led to our goal of utilizing an ensemble approach for CNV detection that performs at an optimized balance of sensitivity and precision compared with other combinations of callers and tested parameters (Supplementary Figure 2).

### Detection of common and rare coding CNVs

**Fig. 2:**
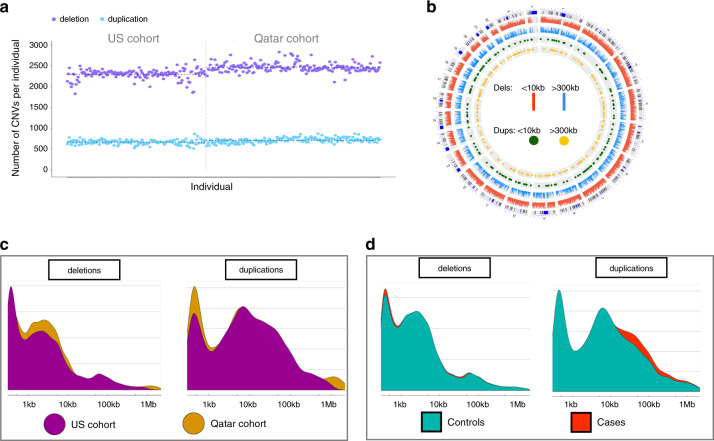
Copy-number variation (CNV) call set. (**a**) Total ascertained CNVs per individual in the respective cohorts (purple = deletion; blue = duplication). All samples included for common and rare variant analyses exhibited comparable read depth and insert size profiles. (**b**) Circos plot representing small (<10 kb) and large (>300 kb) CNVs observed in our cohorts (red = deletions under 10 kb; blue = duplications under 10 kb; green = deletions over 300 kb; yellow = duplications over 300 kb). (**c**–**d**) CNV size distributions of all deletions or duplications are nearly identical between US and Qatar cohorts (**c**) and between all cases and controls combined (**d**).

Our ensemble CNV approach detected a mean of 2,389 deletions and 692 duplications per genome with only slightly more CNVs in the Qatar cohort, consistent with similar observations in population substructures including African ancestry (Fig. 2a). Although CNV sizes spanned from several hundred base pairs to several megabases (Mb) in size, no statistically significant difference in the CNV size distribution was seen between cases and controls (*P* = 0.548) (Fig. 2d). In addition to our CNV analyses, we deployed a computational pipeline in a subset of our NTD cases and controls to analyze and compare the distributions of MEIs. Aside from encompassing over 50% of human genomes, genomic variation caused by Alu, SVA, and LINE-1 (L1) elements are associated with risks for multiple human diseases.^31^ We investigated these abundant forms of genomic variation to ascertain whether they contribute to NTD cases disproportionately more than in controls. Finding no significant difference in the distribution of the number of MEIs between our SB cases and controls (*P* = 0.491), our data did not support a role of mobile elements in SB (Supplementary Figure 3).

**Fig. 3:**
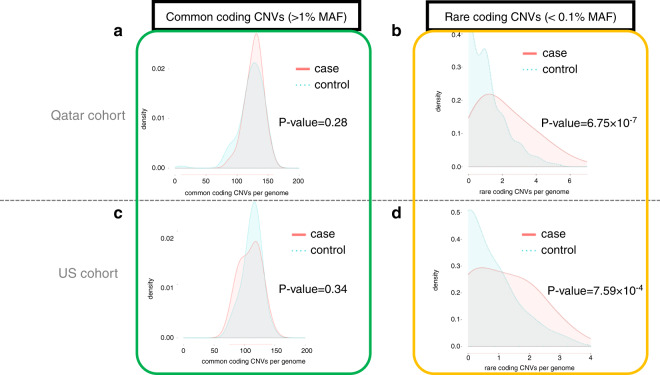
Burden of coding copy-number variants (CNVs) in spina bifida (SB) cases vs. controls. (**a**, **b**) Relative distributions of common and rare coding CNVs in the Qatar case–control cohort. (**a**) The frequency distribution of common coding CNVs does not significantly differ between cases and controls (*P* = 0.28). (**b**) In contrast, a significant enrichment of rare coding CNVs is observed in cases compared with controls (*P* = 6.75 × 10^−7^). (**c**–**d**) Common and rare coding CNV case–control comparison in the US cohort. (**c**) Common CNVs per genome distributions do not significantly differ in cases vs. controls (*P* = 0.34). (**d**) However, a significant enrichment of rare coding CNVs is found in cases compared with controls in this group (*P* = 7.59 × 10^−4^). MAF minor allele frequency.

We sought to identify CNVs with greatest potential to disrupt gene function and thus contribute to NTD pathophysiology. Therefore, our analyses focused on coding CNVs, which we defined as those variants that overlap a coding exonic region by at least one nucleotide. We analyzed common and rare CNVs separately under the assumption that while common CNVs may modify genetic risk, rare CNVs are under more selective pressure and are thus inherently more deleterious. Among common coding CNVs (>1% minor allele frequency [MAF] in population genomic databases), we observed no significant difference between cases and controls in our two cohorts (Fig. 3a, c). In contrast, among the rare coding CNVs, which we defined as those <0.1% MAF, we observed a statistically significant enrichment in cases compared with controls in both the US cohort and the Qatar cohort (*P* = 7.59 × 10^−4^ and *P* = 6.75 × 10^−7^, respectively) (Fig. 3b, d). As with the common coding CNVs, variant size was not a significant factor between cases and controls for the rare coding CNVs (*P* = 0.917), including when we stratified by each cohort (Qatar: *P* = 0.914; US: *P* = 0.652). Moreover, the distributions for the coding CNVs that were within the 0.1–1% MAF range did not reach significance in either cohort (Qatar: *P* = 0.182; US: *P* = 0.234). More CNVs in the Qatar SB case–control cohort were classified as rare compared with the US SB case–control cohort, presumably due to less Middle Eastern representation in existing population databases. This, however, does not alter the significance of the burden analysis as each case–control comparison was ancestry-matched.

### Rare coding CNVs and potential functional significance

**Fig. 4:**
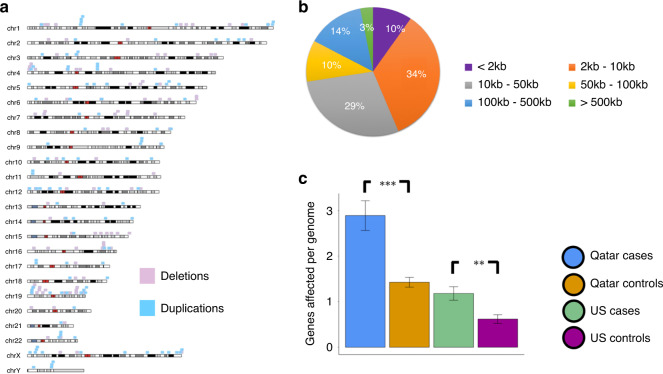
Characterization of detected rare coding copy-number variants (CNVs) by locus, size, and genic content. (**a**) Chromosomal location of rare coding CNVs found in spina bifida (SB) cases shows broad distribution across the genome. (**b**) Size breakdown of the rare coding CNV call set in SB cases. Observed rare coding CNVs from both cohorts are categorized into six bins corresponding to the detected CNV size. (**c**) Comparison within cohorts of the mean value of genes affected by rare coding CNVs per genome in cases compared with controls **p < 0.01; ****p < 0.0001.

**Fig. 5:**
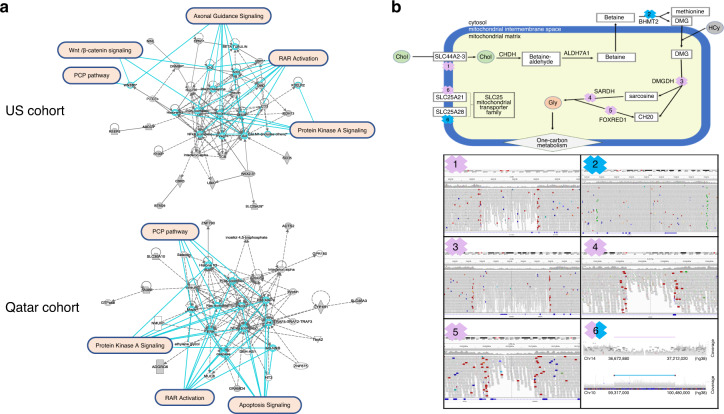
Rare copy-number variants (CNVs) in spina bifida (SB) cases participate in SB-relevant pathways and affect various aspects of one-carbon metabolism. (**a**) Signaling pathways enriched in cases by gene disrupting, rare coding CNVs detected in the US and Qatar cohorts. Shown are Ingenuity Pathway Analysis (IPA) plots. Several of these pathways in both cohorts emerged as enriched in SB cases. PCP planar cell polarity, RAR retinoic acid receptor. (**b**) Metabolic processes of choline transport and one-carbon metabolism that are disrupted by rare coding CNVs found in SB cases are labeled 1–6. Purple = deletions; blue = duplications. Their corresponding CNVs are represented in the lower panel in (**b**) using the Integrative Genomics Viewer (IGV) (for CNV 1–5) and samplot (for CNV 6). Chol choline, Hcy homocysteine, Gly glycine.

Chromosomal locations of rare CNVs seen in SB cases as well as size breakdowns of rare coding CNVs in our cohort are shown in Fig. 4. Slightly more than half of the rare coding CNVs detected were gene disrupting deletions compared with duplications (52.75% vs. 47.75%), which was true for both cohorts (Supplementary Figure 4). The mean number of genes per genome that were disrupted by rare coding CNVs was significantly higher in SB cases relative to controls in both cohorts (*P* < 0.01) (Fig. 4c). The genes affected by rare CNVs in our human SB cases were subjected to pathway analysis using IPA software and, in both cohorts, defined several canonical signaling pathways associated with NTDs, including retinoic acid, protein kinase A (PKA), PCP, and, in the US cohort, WNT/ßcatenin pathways (Fig. 5a). However, these pathways did not reach statistical significance after correction for multiple hypothesis testing. Taking another approach, overrepresentation of genes perturbed by rare coding CNVs in KEGG pathways did suggest potential disruption for cAMP signaling (*P* = 1.18 × 10^−3^), though at a relaxed false discovery rate (FDR) of 0.176 (Supplementary Figure 5).

Within metabolic pathways, we found rare coding CNVs in genes serving several cellular and mitochondrial processes, including choline transport and catabolism, which are closely associated with NTD formation (Fig. 5b). Choline’s oxidation to betaine within the mitochondria provides a link to folate-dependent, one-carbon metabolism, suggesting that the observed gene disrupting variants are likely to contribute to SB risk. For example, *SLC44A2* and *SLC44A3* belong to the SLC44 family of transporters that exhibit choline transmembrane transporter activity. *BHMT2*, which has been associated with NTDs,^32^ functions as a methyl transferase to catalyze the transfer of a methyl group from betaine to homocysteine. Homocysteine is an intermediate of methionine metabolism and has also been linked with NTD risk in a number of studies.^33^ *DMGDH* is directly involved in the catabolism of choline to form sarcosine and is an essential enzyme in the glycine cleavage system, an important mitochondrial process known to harbor genetic risk variants for SB.^34^ The visual representations of the CNVs we highlight in the metabolic pathway in Fig. 5b are also included as Supplementary Figure 6 to aid in clearer inspection of the reads near the breakpoints of the CNVs. These visualizations display consistent genomic read signatures for each of these CNVs, and was part of the manual curation and validation process. Additional potentially SB-relevant rare CNVs that were detected only in cases are listed in Supplementary Table 1. This includes partial duplications we identified in SB cases impacting *PARD3*, an established NTD risk gene^35^ that directs polarized cell growth and affects asymmetric division, as well as *VAV2*, which has several roles in actin dynamics and cytoskeletal remodeling. These cell polarity and cytoskeletal processes are increasingly associated with NTD risk and have led to the discovery of additional novel candidate genes.^36^ For the samples harboring relevant CNVs in which DNA remained available, qPCR was performed to experimentally validate our in silico findings and we include examples as Supplementary Figure 7 (primer designs provided in Supplementary Table 2). Finally, Supplementary Table 3 shows the positions of qualifying rare exon disruptive CNVs that fell within IPA-defined signaling pathways in both Qatar and US cohorts and overlap those CNV regions previously identified^37^ as having an impact on human neurodevelopment. Five of the CNVs so identified in our study overlapped CNVs associated with autism spectrum disorder (ASD), attention deficit–hyperactivity disorder (ADHD), obsessive compulsive disorder (OCD), or schizophrenia and were considered by those investigators^37^ to be clinically relevant according to American College of Medical Genetics and Genomics (ACMG) guidelines.

## DISCUSSION

Here we report a systematic interrogation of the CNV landscape in human SB and find an increased burden of rare CNVs directly affecting coding nucleotides. Integrative CNV pipelines from GS data provide better resolution for variant detection over other conventional methods including array comparative genomic hybridization (aCGH) and ES. In particular, 44% of the rare coding CNVs in our cohorts were less than 10 kb in size, and many would have gone undetected using array-based or ES assays. The ensemble approach used here for CNV discovery in SB cases detected rare CNVs disrupting genes not previously associated with SB, but that participate in pathways of biological significance for neurulation. Moreover, the observation of rare CNVs in some known NTD risk genes and pathways, including one-carbon metabolism, reinforces the validity of this strategy.

Digenic variants have been observed in a number of mouse models of NTDs^38^ as well as in human studies, suggesting synergistic deleterious effects of variants in genes involved in folate metabolism^39^ or in PCP component genes.^40^ While larger cohorts will be needed to reach statistical power necessary to pinpoint specific gene combinations indicative of individual risk, an oligogenic or polygenic model of SB risk is gaining traction and should be considered when evaluating genomic contribution. Indeed, we previously reported evidence using predicted deleterious exonic SNPs genome-wide to propose an omnigenic model of NTD risk. This threshold model of NTD risk was based on accumulation of singleton loss-of-function variants (SLoFVs), regardless of the genes harboring these variants.^41^ In the current study, demonstration of the enrichment of rare gene disrupting CNVs in cases supports extending this threshold burden model of SB risk to include these SVs. That the burden of rare coding CNVs is present in both SB cohorts interrogated in this study supports the notion that our results are not due to effects of population stratification that could confound the interpretation of rare CNVs.

The overlap of the rare gene disrupting CNVs identified here contributes to a resource that may one day enhance clinical utility as it may soon be possible to examine the GS of an infant with SB for prognostic indicators. For example, SB individuals with rare CNVs disrupting genes previously associated with neurodevelopmental disorders may alert to the need for early and vigorous intervention to optimize cognitive development and communication skills in addition to physical therapy. There is much more to be explored, as our high-confidence detection approach to identify rare CNVs using short-read GS data almost certainly underestimates the contribution of structural genome variation to SB risk. In the future, multiplatform approaches and integration of long-read sequencing technology promise to enable detection of more SVs per genome. Clearly, SVs, including CNVs, are an understudied form of genomic variation in SB that warrants further investigation.

Our analyses of the CNV landscape in our SB cohorts underscore that candidate gene approaches limited to exons do not capture the full scope of genomic variation contributing to risk. Functional experiments will ultimately be critical for vetting genomic variants as they relate to NTD predisposition. Nevertheless, interrogating CNVs genome-wide expands the repertoire in SB research of variants and genes with potential to contribute to genetic and gene–environment interactions that interfere with neurulation. Evidence is accumulating to support the view that threshold burden models of SB pathogenesis and GS analyses will achieve a more thorough characterization of the genetic architecture of NTDs.

## Supplementary information

Supplementary Information

## Data Availability

Data pertaining to specific variants generated during the downstream analyses, which support the findings of this study, are available upon request to the corresponding author (M.E.R.). De-identified data will be made available upon request.
