# Supplementary material for: Genome-wide investigation identifies a rare copy-number variant burden associated with human spina bifida
Source: Genet Med. 2021 Mar 8;23(7):1211–8. doi: 10.1038/s41436-021-01126-9 (PMC8257499; doi:10.1038/s41436-021-01126-9)
Supplement: Supplementary file 1 — Supplementary Information [file 41436_2021_1126_MOESM1_ESM.pdf]

Gene Symbol	Chromosome	CNV Start	CNV End	CNV Size (bp)	CNV Type	Gene region	Role / Function
DMGDH	5	79016771	79027799	11028	DEL	intron12-intron14	mitochondrial catabolism of choline; catalyzes the oxidative demethylation of dimethylglycine to form sarcosine
FOXRED1	11	126269114	126271073	1959	DEL	exon1-intron1	encodes a mitochondrial protein that contains a FAD-dependent oxidoreductase domain
SH3GL3	15	83447969	83496803	48834	DEL	intron1-intron2	CNS development, signal transduction, clathrin mediated endocytosis; associated with congenital cerebellar ataxia and Huntington disease
PARD3	10	34811101	34955900	144799	DUP	txStart-intron1	asymmetrical cell division and polarized cell growth (known NTD risk gene)
PGGT1B	5	115224330	115232674	8344	DEL	intron5-intron6	motor neuron axon outgrowth; interacts with RHOB and CDC42
SLC44A2	19	10620876	10626102	5226	DEL	txStart-intron1	dietary choline transporter to synthesize betaine
VAV2	9	133990725	134020294	29569	DUP	txStart-intron1	signal transduction; several roles in cytoskeletal and actin dynamics; expressed in most tissues
DOCK10	2	225033901	225042300	8399	DEL	exon1-intron1	guanosine nucleotide exchange factors for Rho GTPases; interacts with CDC42

Supplementary Table 1. Rare, gene disrupting CNVs present only in cases that have potential for biological significance for neurulation. Shown are genic location of the CNV and known biological function of the gene disrupted.

Primers	Sequence (5' to 3')	Size of amplicon
DMGDH-F	CAGGGAGGCACATCAAAAA	151 bp
DMGDH-R	TCCAGACTCACAGGCTCTGA	
SH3GL3-F	GGACCATGGCAAACTGAGAT	152 bp
SH3GL3-R	GACTCGTAAACCCTGGGGAT	
PARD3-F	ACTGCAGAGAAAGCCTTGGT	130 bp
PARD3-R	CACACTTTTCCCCACCAGAT	
DOCK10-F	GGGAAATTCAAGAGCCCAAT	136 bp
DOCK10-R	TCCAGACTCACAGGCTCTGA	

Supplementary Table 2. List of gene-specific primers used for qPCR.

Chromosome (chr)	Start Position	End Position	CNV Size (bp)	CNV Type	NDD Cohort Overlap
chr2	47161001	47363000	202000	DUP	
chr2	212520978	212598541	77563	DUP	
chr5	58964501	59009100	44599	DUP	
chr5	129407112	129601002	193890	DUP	ASD
chr5	240783	607807	367024	DUP	ADHD, ASD, OCD, SCZ*
chr5	66078401	66079900	1499	DUP	
chr6	38597553	38605072	7540	DEL	
chr7	6423456	6605006	181551	DEL	ASD
chr7	70769343	71410241	640898	DUP	ADHD, ASD*, SCZ
chr8	99101150	99103517	2320	DEL	
chr9	133990725	134020294	29569	DUP	ASD
chr10	99317001	100480000	1.16E+06	DUP	ASD
chr10	34811101	34955900	144799	DUP	ADHD, ASD
chr11	31514249	31534087	19838	DEL	ASD
chr12	110607001	110741000	134000	DUP	
chr12	79721001	79828000	107000	DUP	
chr13	19177560	19197244	19684	DEL	
chr14	36672883	37212020	539138	DEL	ASD
chr15	67196115	67462384	266269	DEL	ASD, OCD
chr16	83165419	83188058	22654	DEL	
chr16	76464757	76728295	263538	DUP	ASD, OCD
chr18	79439001	79542000	103000	DUP	ASD*
chr18	55612876	55758301	145426	DEL	ASD, SCZ
chr18	41512637	42123625	610988	DUP	ADHD, ASD
chr19	4155994	4166977	10983	DUP	
chr19	3121001	3124100	3099	DUP	
chr19	4524505	4545286	20781	DEL	
chr20	25491469	25493839	2349	DEL	ASD
chrX	154881001	155372000	491000	DUP	ADHD*, ASD, OCD, SCZ
chrX	1096026	1382360	286334	DUP	
chrX	6535001	8169000	1.63E+06	DUP	ADHD*, ASD, OCD*, SCZ
chrX	8627001	8738000	111000	DUP	ADHD, ASD, OCD
chrX	31743206	31848338	105133	DEL	ADHD, ASD

Supplementary Table 3. Partial list of rare exon disruptive CNVs in this study. Shown are detected rare CNVs that impact either established NTD pathways shared by both Qatar and US cohorts (highlighted in Fig. 5a) or that overlap established human neurodevelopmental disorder (NDD) genes. Variants are shown by chromosomal location, size and type. NDD cohort overlap specifies the neurodevelopmental disorder in which the rare coding CNV found in our SB cases overlaps with a CNV detected in a NDD cohort (Zarrei et al., 2019, NPJ Genomic Medicine, 4:26). * signifies that the CNV from our study overlaps a NDD CNV previously deemed to be clinically relevant by ACMG guidelines. ADHD=attention deficit hyperactivity disorder; ASD=autism spectrum disorder; OCD=obsessive compulsive disorder; SCZ=schizophrenia.

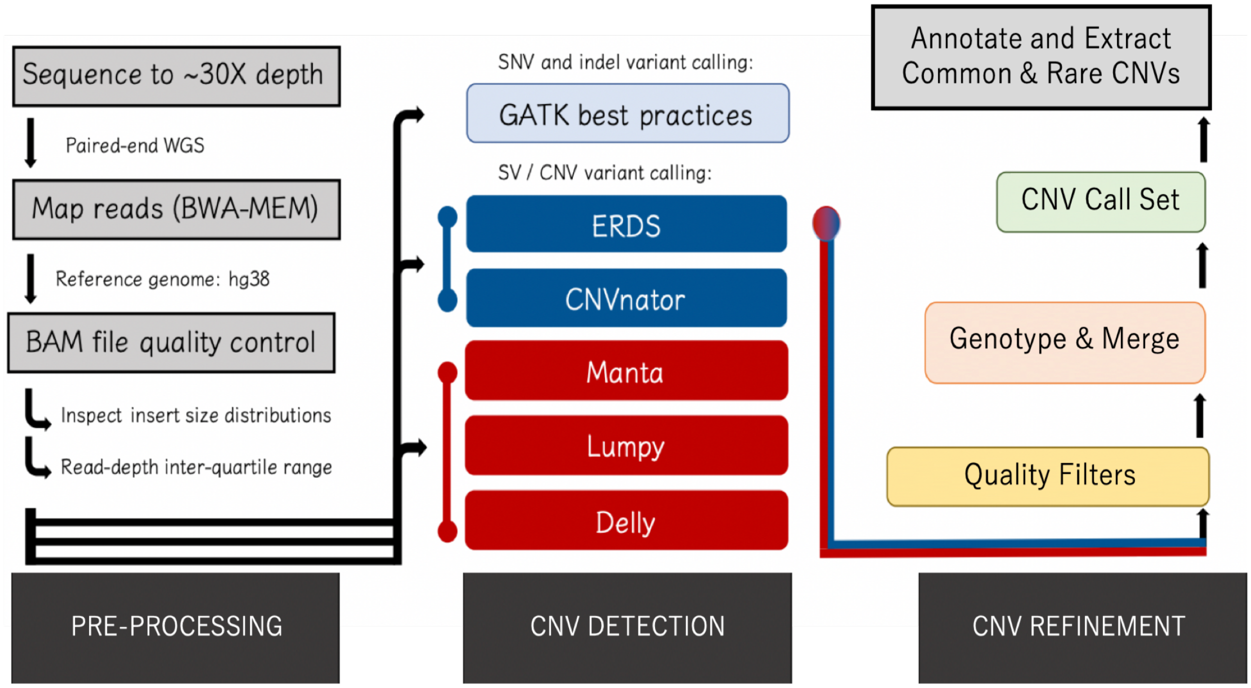

Supplementary Figure 1. Workflow schematic for CNV detection in our spina bifida cohorts. Pre-processing, CNV detection and call refinement are core components of the computational methodology in our study to obtain high-quality CNVs from WGS data. For the ensemble approach used for CNV detection, the evidence from read depth information is incorporated using the algorithms in blue (ERDS and CNVnator) and the split-read and read pair evidence is integrated using the algorithms in red (Manta, Lumpy and Delly).

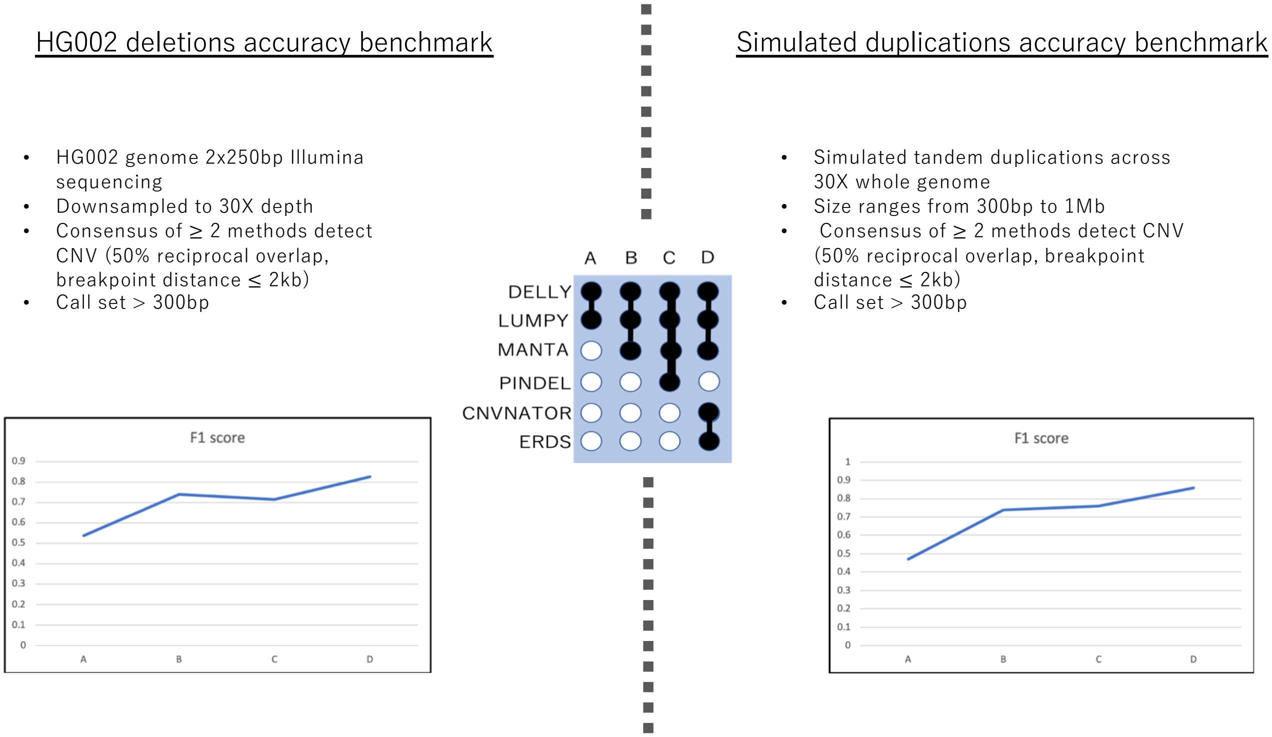

Supplementary Figure 2. Benchmark and comparison testing for several ensemble approaches for CNV detection on real (HG002) and simulated (30X) genomes. CNV detection accuracy was measured using 50% reciprocal overlap.

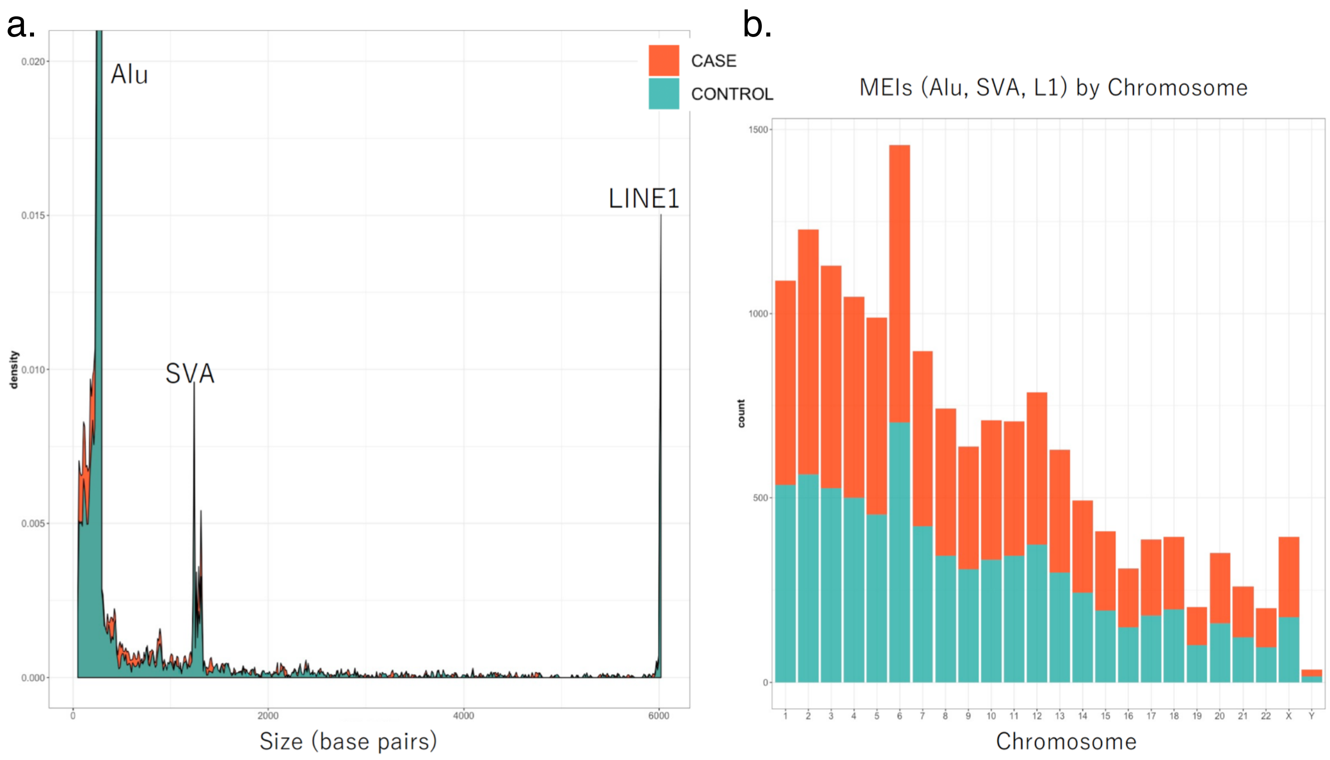

Supplementary Figure 3. Characteristics of mobile element insertions (MEIs) observed in our US spina bifida cohort. (a) Density plots of MEIs (Alu, SVA and L1) detected in US cases and controls. (b) Counts of Alu, SVA and L1 elements per chromosome for US cases and controls.

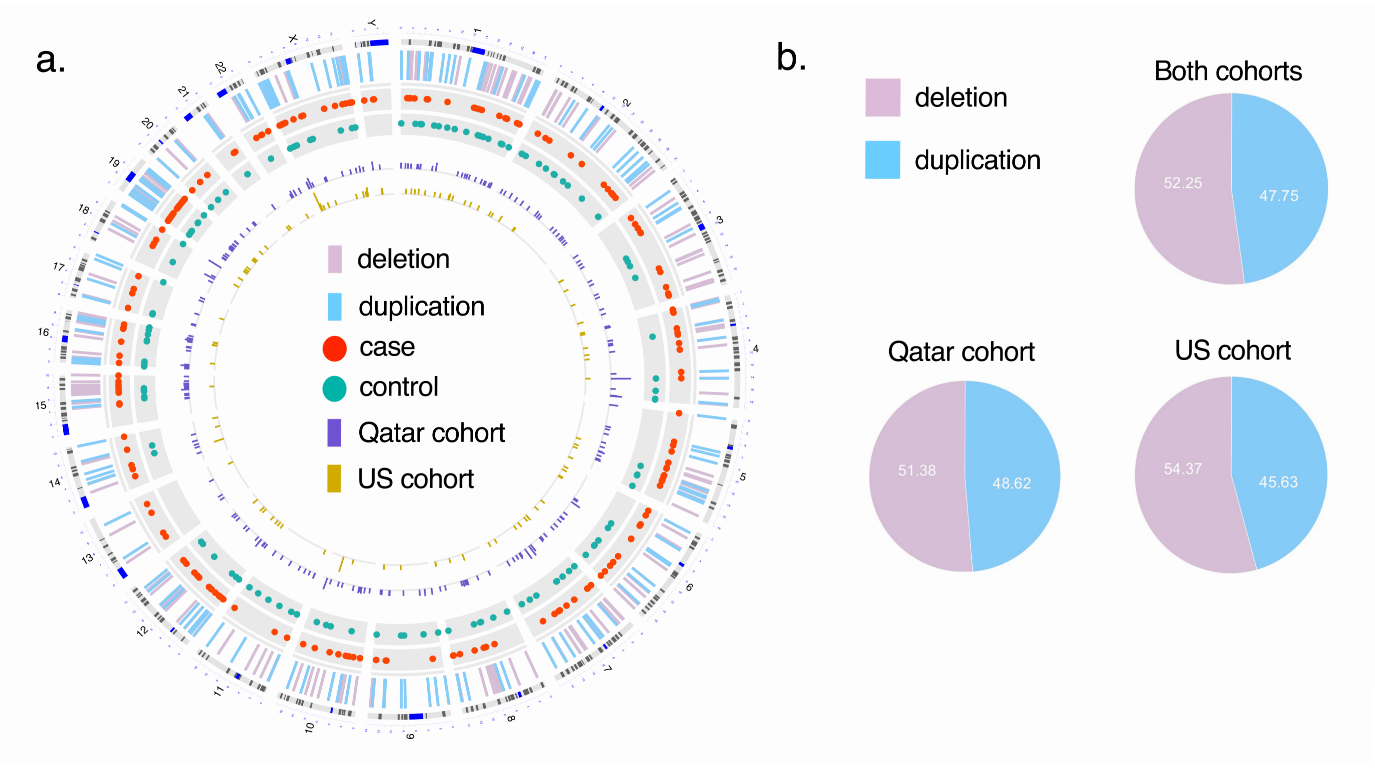

Supplementary Figure 4. Rare coding CNV chromosomal locations and comparison of genetic losses and gains in each cohort. (a) Circos plot depicting chromosomal locations of rare coding CNVs detected in each population cohort by case status. (b) Proportions of rare coding deletions (pink) and duplications (blue) in each population cohort were consistent.

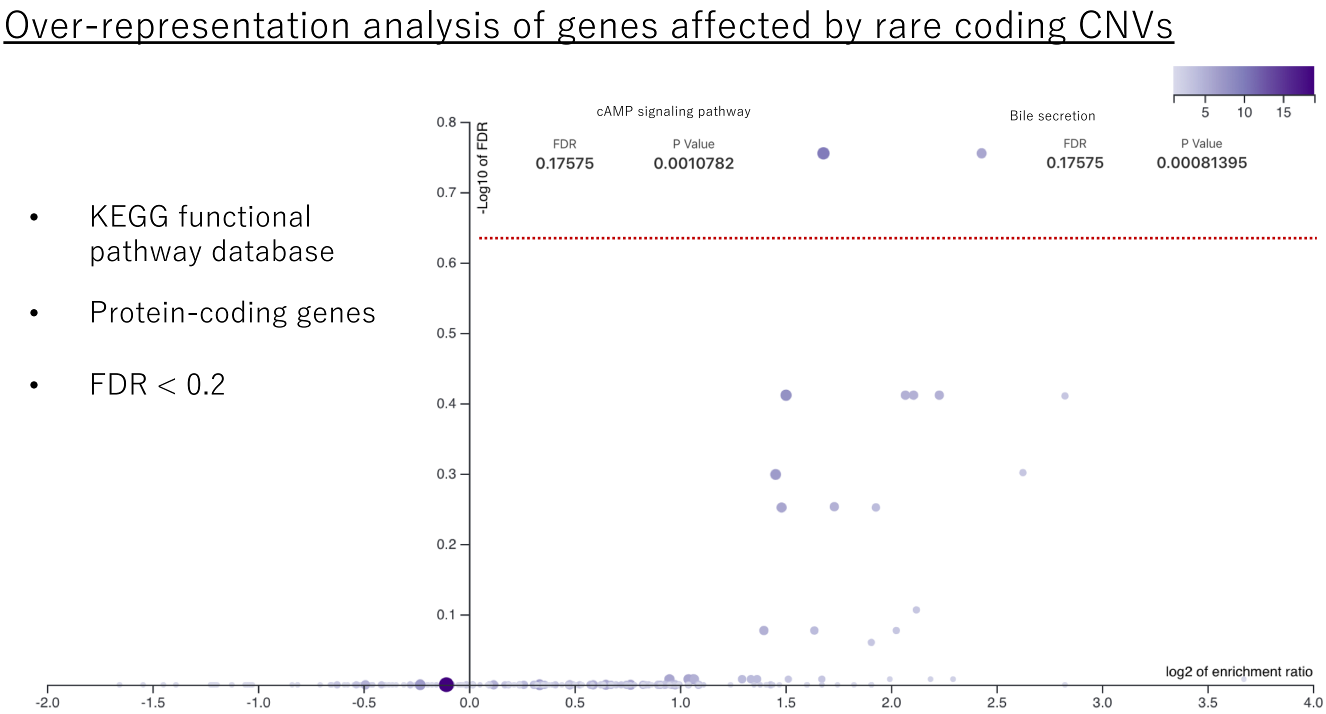

Supplementary Figure 5. Over-representation analysis of the protein-coding genes affected by rare coding CNVs in SB cases in the study. Two pathways using the KEGG functional pathway databases are significantly enriched when screened for a false discovery rate (FDR) under 0.2 (cAMP signaling pathway and bile secretion).

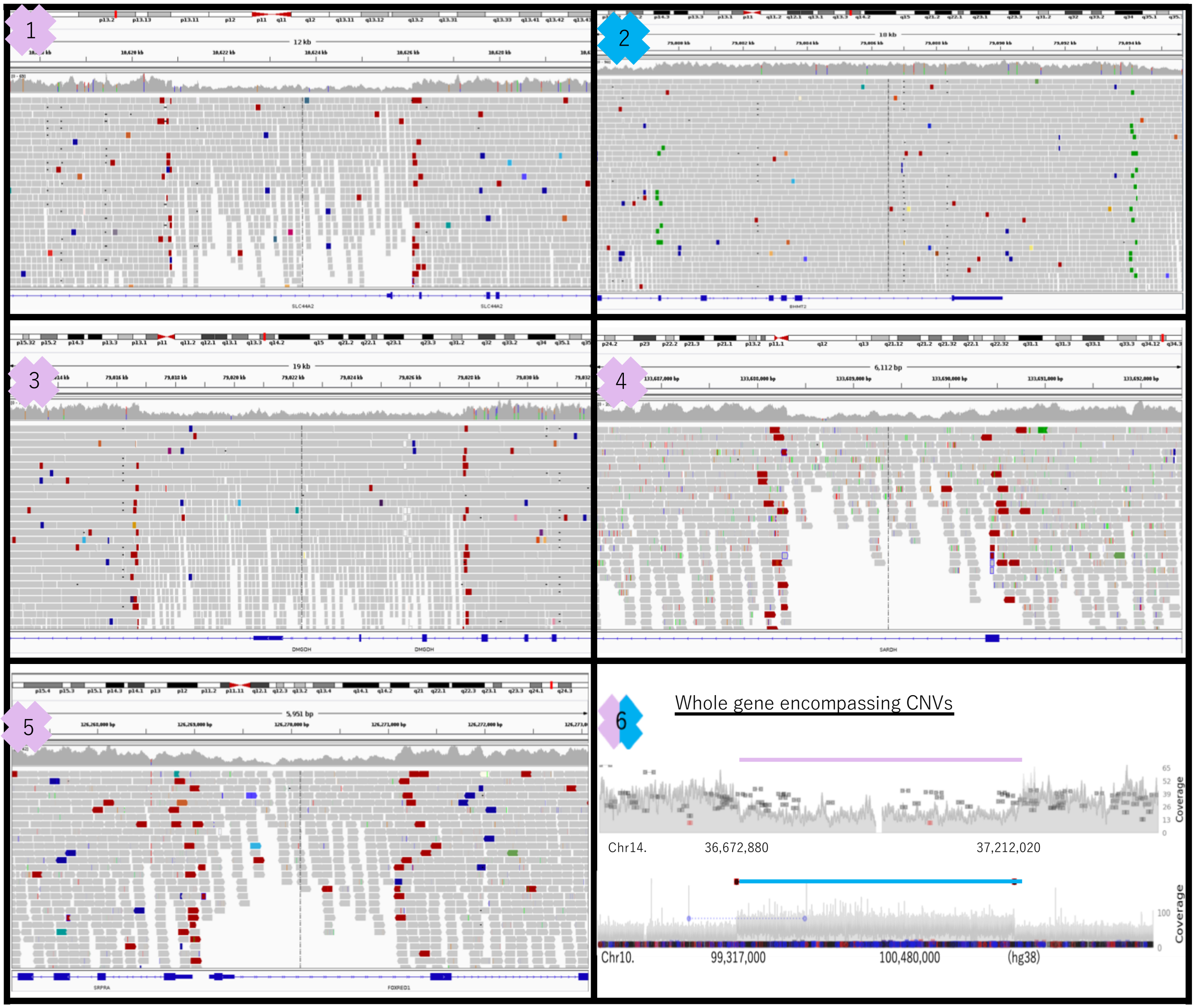

Supplementary Figure 6. Visual representations of the CNVs highlighted in Figure 5b. Integrative Genomics Viewer (IGV) images show consistent genomic signatures in the detected CNVs as well as the read information proximal to CNV breakpoints. Labels: 1=SCL44A2; 2=BHMT2; 3=DMGDH; 4=SARDH; 5=FOXRED1. The samplot (labeled 6) displays whole gene encompassing CNVs (deletion=SLC25A21; duplication=SLC25A28). Purple=deletions; blue=duplications.

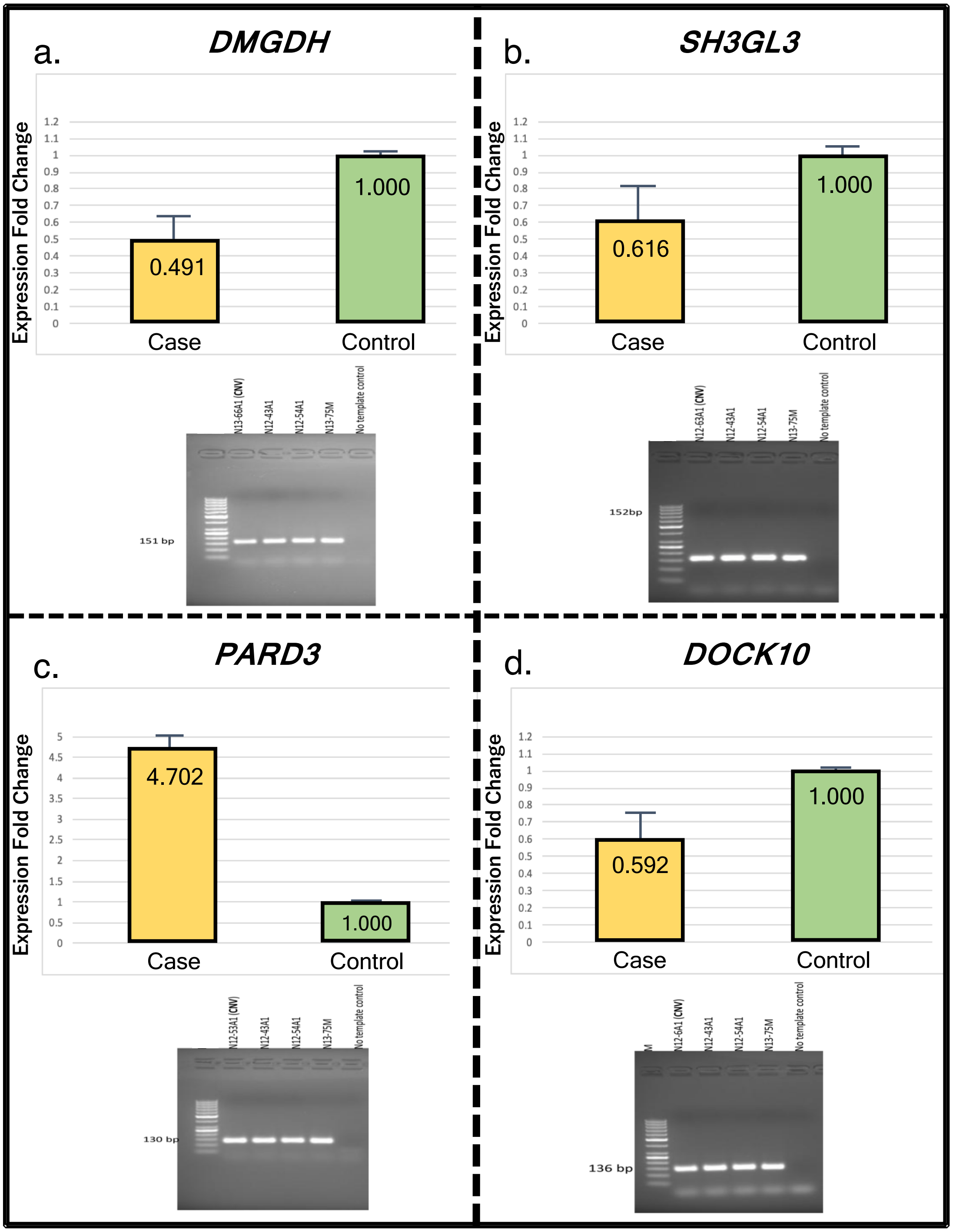

Supplementary Figure 7. qPCR validation of select rare coding CNVs. (a-d) Fold change in levels of *DMGDH*, *SH3GL3*, *PARD3* and *DOCK10* compared to three unaffected controls as determined by qPCR. Primer designs are in Suppl. Table 2. All samples were run in triplicate. The fold changes in expression were calculated using the $2^{-\Delta\Delta CT}$ method and estimates outside of the 1-3 expression fold change range were considered to be CNVs (<1=deletion; >3=duplication).
